# Translating food safety and nutrition policy into school-based practice: Evidence from Indonesian primary schools

**DOI:** 10.1016/j.puhip.2026.100844

**Published:** 2026-08-20

**Authors:** Cica Yulia, Ali Khomsan, Rina Rifqie Mariana, Ade Chandra Iwansyah, Delita Septia Rosdiana, Desiani Rizki Purwaningtyas, Desyane Ramadhina Sari

**Affiliations:** a Culinary Arts Education Study Programme, Universitas Pendidikan Indonesia, Bandung, Indonesia; b Nutrition Science Study Programme, IPB University, Bogor, Indonesia; c Culinary Arts Education Study Programme, Universitas Negeri Malang, Malang, Indonesia; d Research Center for Food Technology and Processing, National Research and Innovation Agency, Indonesia; e Department of Nutrition, Universitas Pendidikan Indonesia, Bandung, Indonesia; f Technology and Vocational Education, Universitas Pendidikan Indonesia, Bandung, Indonesia

**Keywords:** Food safety education, Nutrition education, Primary schools children, Public health practice, Policy implementation

## Abstract

**Objectives:**

Foodborne diseases and poor nutrition remain major public health challenges, particularly among school-aged children. Although schools are recognised as key settings for promoting healthy eating and food safety behaviours, evidence to guide integrated school-based programmes in Indonesian primary schools remains limited. This study aims to explore current conditions, existing programmes, implementation challenges, and future policy directions to inform the development of an integrated school-based education model.

**Study design:**

Qualitative exploratory study using focus group discussions and semi-structured in-depth interviews.

**Methods:**

The study was conducted between April and November 2025 in urban and rural settings in Indonesia. Data were collected through focus group discussions and in-depth interviews involving 21 stakeholders from the education, health, and food security sectors, including school principals, nutrition experts, curriculum specialists, and teacher working groups. Audio recordings were transcribed verbatim and analysed thematically using NVivo 12 through iterative coding, categorisation, and theme development.

**Results:**

Schools demonstrated institutional readiness and cross-sectoral support for food safety and nutrition education. However, implementation remained fragmented due to the absence of standardised curricula, structured learning modules, and assessment tools, alongside challenges related to informal food vendors, inconsistent canteen hygiene, and limited handwashing facilities. Behavioural change was consistently identified as the primary indicator of programme success. These findings informed the development of the GREENEDU NEXUS model, comprising five interconnected pillars: standardised curriculum, experiential learning, safe and sustainable canteens, green behaviour and food waste reduction, and partnership and advocacy.

**Conclusions:**

The findings suggest that Indonesian primary schools are receptive to an integrated, whole-school approach to food safety and nutrition education. Strengthening curriculum standardisation, improving school food environments, and reinforcing cross-sectoral collaboration are essential in translating policy into sustainable public health practice.

1 **What this study adds**

● Provides qualitative evidence on current conditions and implementation gaps in school-based food safety and nutrition education in Indonesia.
● Highlights stakeholder perspectives on institutional readiness, challenges, and success indicators for effective programme implementation.
● Offers an evidence-informed foundation for developing an integrated, sustainability-oriented education model for primary schools.

2 **Implications for policy and practice**

● Food safety and nutrition education should be institutionalised within formal curricula and school food governance through standardised modules and minimum infrastructure standards.
● Implementation should prioritise behavioural empowerment supported by experiential learning and safe, sustainable school food environments.
● Pilot implementation and rigorous evaluation are needed to assess impacts on student behaviours and school food systems.

## 1 Introduction

Food safety and nutrition are strategic global issues with direct implications for public health and the quality of human resources. [[1], [2], [3]] Beyond improving quality of life, food safety assurance plays a critical role in reducing health risks associated with microbial contamination and hazardous chemical exposure, which are collectively referred to as Foodborne Diseases (FBDs). [4] Although FBDs can affect all population groups, children, older adults, pregnant women, and immunocompromised individuals face a disproportionately higher risk of severe health consequences. [5] The global burden of FBDs remains substantial; data from the World Health Organization indicate that 31 major hazards were responsible for approximately 600 million FBD cases worldwide in 2010, resulting in 33 million Disability-adjusted Life Years (DALYs) and 420,000 deaths. [6,7] Diarrheal diseases account for more than half of all FBD-related infections, [8] with Indonesia reporting the highest prevalence of diarrhea among five Southeast Asian countries at 18.21%, surpassing Cambodia, Myanmar, the Philippines, and Timor-Leste. [9] School-aged children are particularly vulnerable, as diarrhea remains the second leading cause of child mortality globally. [10,11]

In the Indonesian context, food safety challenges within school environments persist and continue to pose serious public health concerns. Evidence shows that 98.9% of school-aged children consume ready-to-eat foods, increasing their exposure to unsafe food practices and contributing to recurrent food poisoning incidents in schools. [12] Notable cases include food poisoning among elementary school students in East Java after consuming candy-shaped snacks in 2022, [12] 2897 poisoning cases reported in Bandar Lampung with children aged eight years being the most affected, [13] and multiple food poisoning outbreaks or *Kejadian Luar Biasa* (KLB), [14] and incidents involving approximately 30 elementary school students in Sukabumi in early 2024. [15] These events have been largely attributed to improper food handling practices and limited consumer awareness regarding food safety and quality. [16]

Despite the growing body of research on school-based food safety interventions, [[17], [18], [19]] important gaps remain. Evidence on how food safety and nutrition education can be systematically integrated into primary school curricula in developing countries is still limited, particularly in contexts with diverse resources and local constraints. In addition, although green economy approaches in food systems have gained attention, [20,21] their application within school-based food safety and nutrition education has rarely been explored. Furthermore, comprehensive qualitative needs assessments that incorporate multi-stakeholder perspectives, including education authorities, health sectors, agriculture offices, and curriculum experts, remain scarce in low- and middle-income countries. Existing interventions also tend to emphasize knowledge acquisition rather than sustained behavioral change and environmental responsibility, [22,23] highlighting the need for a holistic, context-specific educational model that integrates food safety, nutrition, and green economy principles.

**Fig. 1:**
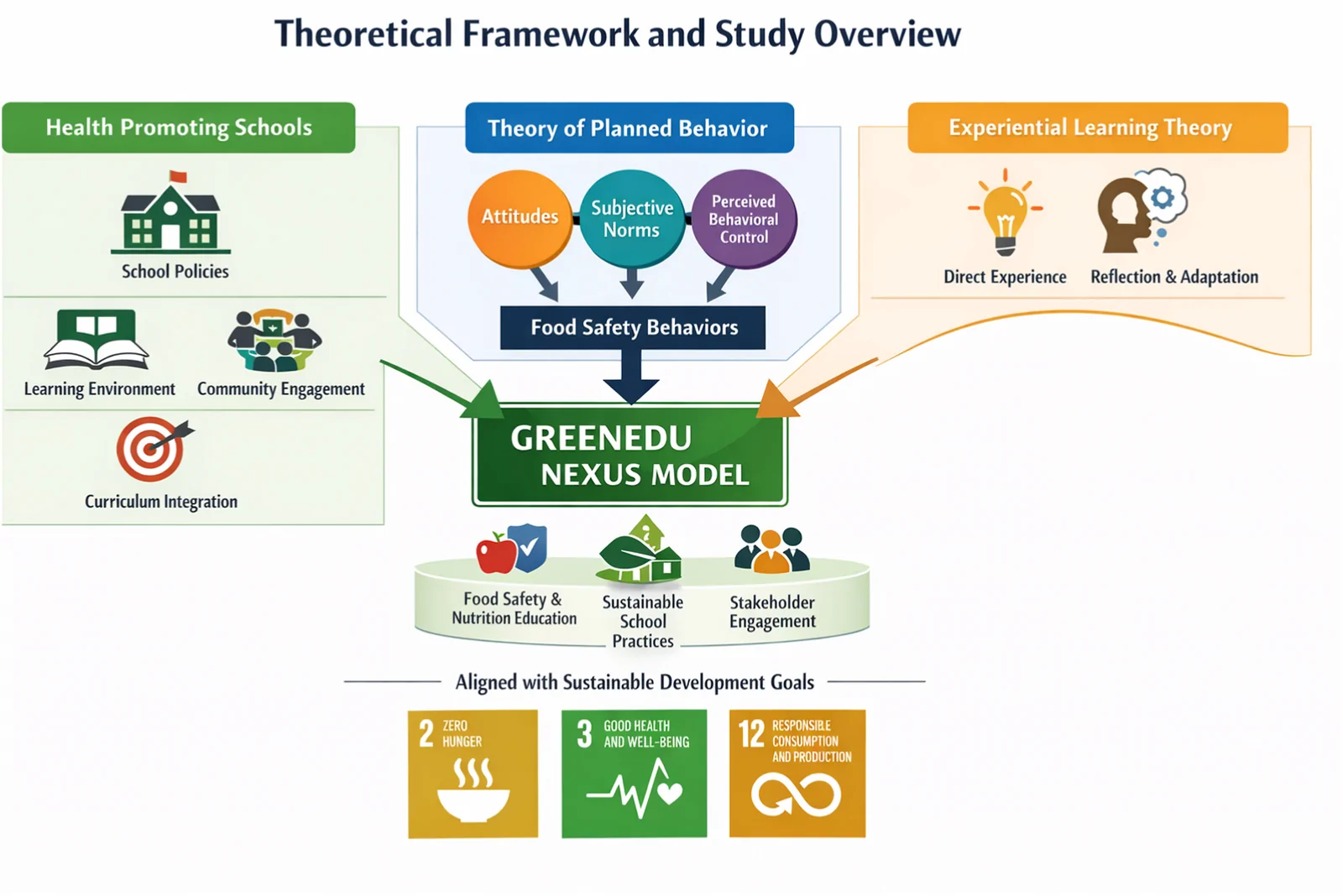
Theoritical framework and study overview.

This study was theoretically informed by the Health Promoting Schools (HPS) framework, which emphasizes the integration of health education across school policies, physical environments, learning processes, and community engagement, [24] the Theory of Planned Behavior, which explains how attitudes, subjective norms, and perceived behavioral control shape food safety behaviors, [25] and Experiential Learning Theory, which underscores learning through direct experience and reflection as a pathway to sustainable behavioral change. [26] Guided by these frameworks (Fig. 1), this study aimed to conduct a comprehensive qualitative needs assessment to support the development of an integrated food safety and nutrition education model based on green economy principles for Indonesian primary schools. The assessment focused on existing curriculum integration, stakeholder perceptions, school food policies, and key facilitators and barriers to implementation. The findings were used to inform the development of the GREENEDU NEXUS model, a novel educational framework integrating food safety, nutrition, and green economy principles, aligned with the Sustainable Development Goals (SDGs), particularly SDGs 2, 3, and 12, and tailored to Indonesian primary school contexts.

## 2 Methods

### 2.1 Study design

This study employed a qualitative exploratory design using focus group discussions (FGDs) and semi-structured in-depth interviews to explore stakeholder perspectives on food safety and nutrition education in Indonesian primary schools. The study aimed to gain an in-depth understanding of current practices, implementation challenges, and policy needs to inform the development of an integrated school-based educational model. It was conducted between April and November 2025 in two purposively selected regions in Indonesia: Bandung City, West Java Province, representing an urban setting, and Gunungkidul Regency, Special Region of Yogyakarta, representing a rural setting. These sites were selected to capture contextual variation in urbanisation, socioeconomic conditions, and food system characteristics relevant to school food safety and nutrition.

### 2.2 Sample size and sampling

Purposive sampling with a maximum variation strategy was employed to capture diverse perspectives from key stakeholders involved in food safety and nutrition education across urban and rural settings. Participants were intentionally selected based on their institutional roles, professional expertise, and responsibilities related to school food safety, nutrition, curriculum development, and policy implementation. A total of 21 participants were recruited across two focus group discussions, comprising 10 participants from Bandung City and 11 participants from Gunungkidul Regency. The sample represented key stakeholder groups involved in the planning, implementation, and oversight of school-based food safety and nutrition programmes, including representatives from the Regional Development Planning Agency, District Education Office, District Health Office, Food Security and Agriculture Office, primary school principals, heads of primary school teacher working groups, physical education teacher working groups, nutrition experts, and curriculum specialists. Within each government institution, participants were selected from the divisions most directly responsible for the relevant policy areas, such as primary school curriculum, school health and child nutrition, and food safety.

### 2.3 Data collection

Data were collected between April and November 2025 through two Focus Group Discussions (FGDs), complemented by semi-structured in-depth interviews with participants holding strategic roles or specialised expertise to provide methodological triangulation. FGDs explored shared perspectives on food safety and nutrition education, including curriculum content, existing programmes, policy frameworks, implementation challenges, resource availability, and intersectoral coordination, and lasted approximately 90–120 min. In-depth interviews were conducted individually to obtain more detailed insights into participants' areas of expertise and lasted approximately 45–60 min. All discussions and interviews were audio-recorded with participants' consent, transcribed verbatim in Indonesian, and supported by field notes documenting contextual observations and non-verbal interactions.

### 2.4 Data analysis

Data were analysed using thematic analysis in accordance with Braun and Clarke's six-phase approach: familiarisation with the data, generation of initial codes, development of potential themes, review and refinement of themes, definition and naming of themes, and production of the report using illustrative quotations. [27] Audio recordings from the focus group discussions and in-depth interviews were transcribed verbatim in Indonesian and imported into NVivo 12 (QSR International) for data management and analysis. An initial coding framework was developed deductively based on the study objectives and subsequently refined inductively to accommodate emerging concepts from the data. Members of the research team collaboratively reviewed the transcripts and developed the initial coding framework through discussion and consensus. The coded data were subsequently organised and analysed in NVivo 12 by an experienced qualitative data analyst to support the development of themes and sub-themes. The coding framework, theme development, and interpretation of the findings were continuously reviewed and discussed among the research team to enhance the credibility, consistency, and trustworthiness of the analysis. Throughout the analytical process, the research team remained aware of their potential influence on data interpretation and sought to ensure that the findings were grounded in participants' accounts rather than researchers' assumptions.

## 3 Results

### 3.1 Existing program context in schools

Findings indicated that primary schools had established foundational programs related to food safety and nutrition; however, the scope and quality of implementation varied considerably across settings. Routine canteen monitoring by health authorities was reported, yet its consistency and effectiveness differed between schools, particularly in terms of follow-up and standard procedures. As noted by one participant,

“The Health Department conducts routine canteen monitoring, but the quality is not yet uniform across all schools” (Health Department representative, Bandung).”

In parallel, many schools had actively implemented green economy–related initiatives, such as school gardens, waste banks, and anti–food waste campaigns. These activities functioned as experiential learning platforms that supported environmental awareness and student engagement, as illustrated by a school principal in Gunungkidul:

“School gardens, waste banks, and anti-food waste campaigns are actively conducted.”

Although these initiatives reflected existing institutional capacity and stakeholder involvement, they were largely fragmented and operated without a standardized framework. As a result, program outcomes depended heavily on local leadership and resources, highlighting the need for an integrated and systematic model to align existing practices with shared objectives and quality standards.

### 3.2 Curriculum integration opportunities and gaps

#### 3.2.1 Current integration across subjects

Food safety and nutrition content were found to be embedded across several subjects, including Science or *Ilmu Pengetahuan Alam* (IPA), Physical Education and Sports or *Pendidikan Jasmani Olahraga dan Kesehatan* (PJOK), Environmental Education or *Pendidikan Lingkungan Hidup* (PLH), and Pancasila Student Profile Strengthening Projects. Teachers commonly integrated themes of food hygiene and nutrition into thematic and project-based learning activities. A curriculum specialist from Bandung explained that *“Teachers incorporate food hygiene and nutrition topics into thematic projects.”* Schools also demonstrated curricular flexibility through annual curriculum reviews that allowed adaptation to local characteristics and needs, as reflected by one school principal:

*“Every year we review the curriculum and adjust it to our specific school context”* (KS2).

#### 3.2.2 Gaps in standardization and assessment

Despite this cross-subject integration, substantial gaps were identified in terms of standardization. Schools lacked structured learning modules, clear competency indicators, and specific assessment rubrics for food safety and nutrition education. Consequently, instructional depth and learning outcomes varied widely and were highly dependent on individual teacher initiative. In the absence of standardized materials, food safety topics were often deprioritized relative to other health-related issues perceived as more urgent. Stakeholders consistently emphasized the need for uniform instructional guidance, as noted by a school principal:

“For uniformity, guidance in the form of a module on food safety and nutrition for elementary school students is needed” (KS1).

Analysis of curriculum-related findings highlighted three priority needs: the development of standardized, grade-specific learning modules with explicit learning outcomes and assessment criteria; the inclusion of food safety and nutrition as core competencies supported by formal curriculum documents; and the adoption of behavior-oriented learning strategies aligned with experiential learning principles.

### 3.3 Multi-stakeholder support and engagement

#### 3.3.1 Cross-sectoral collaboration

The findings revealed strong multi-sectoral engagement involving schools, parents, education offices, health departments, and local small and medium enterprises (SMEs). Coordination among agencies in supporting food safety and nutrition education was evident, particularly through joint activities and resource sharing. An education office representative from Gunungkidul stated,

“There is coordination among various parties in food safety education.”

Health departments played a central role by providing monitoring, training, and educational materials, as noted by a school principal:

“We collaborate with the health department for pamphlets, videos, and leaflets.”

This collaborative structure reflected a whole-school approach, emphasizing shared responsibility across institutional and community actors.

#### 3.3.2 Family involvement and variability

Families were recognised as key actors in shaping children's food consumption behaviors, particularly through meal preparation and snack regulation. However, stakeholder accounts highlighted substantial variation in family engagement and nutrition literacy. As one teacher working group coordinator observed, *“The family role is strong, but not consistent across students.”* This variability underscored the importance of strengthening home–school collaboration mechanisms to ensure continuity of safe and healthy eating practices beyond the school environment.

#### 3.3.3 Local SMEs and the food supply chain

Engagement with local SMEs in supplying school food emerged as both an opportunity and a challenge. When accompanied by appropriate training and supervision, these partnerships supported local food systems and green economy objectives. Representatives from the Food Security and Agriculture Office described efforts to provide technical guidance to producers, stating, *“We work together with extension workers and pest observers to reduce pesticide use.*” These findings highlighted the potential role of capacity building within the local food supply chain to support safer and more sustainable school food environments.

### 3.4 The GREENEDU NEXUS model framework

Based on the five-dimensional needs assessment, the GREENEDU NEXUS model was developed as an operational adaptation of the whole-school food systems approach, contextualized for Indonesian primary schools and integrated with green economy principles. The model comprises five interconnected pillars.

The mapping presented in Table 1 demonstrates how the GREENEDU NEXUS model was systematically derived from the empirical findings of the needs assessment. Each pillar responds to a specific implementation challenge identified by stakeholders while collectively addressing the broader need for an integrated school-based approach to food safety and nutrition education. Rather than representing independent intervention components, the five pillars are mutually reinforcing: curriculum standardisation provides a consistent educational foundation, experiential learning translates knowledge into practice, safe and sustainable canteens create supportive food environments, green behaviour and food waste reduction foster sustainable behavioural change, and partnership and advocacy strengthen cross-sectoral coordination to support long-term implementation. This alignment between stakeholder-identified priorities and the proposed intervention framework demonstrates the empirical basis of the GREENEDU NEXUS model.

**Table 1:** GREENEDU NEXUS model: Five pillars framework.

Pilar	Empirical Theme	Main Focus	Key Strategies	Expected Behavioral Outcomes
**1. Standardized Curriculum**	Lack of standardised learning materials and assessment	Integration of food safety and nutrition content into structured learning	Grade-specific modules; behavioral assessment rubrics	Enhanced knowledge; positive attitude formation toward food safety
**2. Experiential Learning**	Stakeholder preference for participatory learning	Hands-on practice and direct experience	Student-led canteen audits; hygiene simulations; school food gardens	Improved food hygiene skills and practices
**3.** **Safe and Sustainable Canteen**	Unsafe school food environment, canteen hygiene, informal vendors	Sanitation and healthy food supply	Canteen certification; nutrition labeling; local food sourcing	Increased access to safe food; reduced foodborne disease risk
**4.** **Green Behavior and Food Waste Reduction**	Emphasis on behaviour change and environmental responsibility	Environmentally-friendly consumption culture	Waste banks; zero-leftover initiatives; composting	Reduced food waste; increased preference for healthy, sustainable foods
**5. Partnership and Advocacy**	Need for stronger cross-sectoral collaboration	Cross-sectoral collaboration	Parent education; local food procurement; multi-stakeholder coordination	Consistent behavior both in and outside school settings

## 4 Discussion

This needs assessment revealed an apparent contradiction between the substantial institutional readiness for integrated food safety and nutrition education in Indonesian primary schools and the fragmented implementation observed across educational settings. While foundational elements such as school health programmes, environmental initiatives, and multi-stakeholder involvement were already in place, implementation remained constrained by persistent structural, governance, and pedagogical gaps requiring systematic intervention. These findings suggest that the primary challenge is not the absence of policy commitment, but the limited institutionalisation of governance mechanisms that translate national policies into coordinated implementation, [28] monitoring, and accountability at the school level. [29] Similar implementation gaps have been reported in school health promotion, where enabling structures exist but lack coherent operational frameworks. [30,31] This implementation gap reflects a common challenge in public health programmes, where policy adoption alone is insufficient without adequate implementation capacity, operational guidance, and cross-sectoral accountability mechanisms. [32] Collectively, these findings indicate that strengthening governance and implementation capacity may be more important than introducing additional policy initiatives. Synthesising insights across programme infrastructure, curriculum integration, stakeholder engagement, implementation barriers, and evaluation priorities informed the development of the GREENEDU NEXUS model, a five-pillar framework integrating food safety, nutrition education, and green economy principles within a whole-school approach.

The cross-curricular integration of food safety and nutrition across subjects such as Science, Physical Education, Environmental Education, and P5 projects highlights curriculum flexibility and teacher agency as key strengths. However, the absence of standardised modules, explicit learning objectives, and validated assessment tools results in integration without systematisation, where content depth, accuracy, and evaluability cannot be assured. Heavy reliance on individual teacher creativity creates substantial variability in student learning experiences, effectively producing a “curriculum lottery” that risks reinforcing disparities between schools and regions. This variability reflects broader structural weaknesses in curriculum governance, where national policy aspirations have not yet been translated into practical implementation guidance, teacher support systems, or standardised evaluation mechanisms. [33] This contrasts with structured, competency-based food safety education models in Taiwan and China, which have demonstrated sustained improvements in students’ knowledge and behaviours. [[34], [35], [36]] Recent evidence further suggests that competency-based school health programmes are most effective when curriculum standardisation is accompanied by teacher capacity building, monitoring systems, and supportive school policies. [37] The Standardised Curriculum pillar of GREENEDU NEXUS addresses this gap by proposing grade-specific modules, clear competency indicators, and behaviour-oriented assessment rubrics, balancing consistency with contextual flexibility in line with effective health education frameworks. [38,39]

Pedagogically, strong stakeholder support for experiential learning approaches, such as hygiene simulations, student-led canteen audits, school gardens, and food preparation activities, underscores their importance in translating knowledge into practice, consistent with experiential learning theory and empirical evidence on behaviour change. [[40], [41], [42]] Evidence from school garden initiatives and youth-led food environment assessments further supports the role of experiential strategies in strengthening food safety competencies and environmental awareness. [[43], [44], [45], [46]] Beyond improving knowledge and skills, experiential learning has also been recognised as a key component of whole-school approaches by connecting classroom learning with the broader school food environment and everyday health practices. [47] While multi-sectoral engagement aligns well with WHO-endorsed Health Promoting Schools and whole-school frameworks, [24] coordination challenges persist due to unclear roles and limited shared accountability. Evidence from whole-school health promotion further indicates that sustainable implementation requires clearly defined governance arrangements, formal coordination mechanisms, and shared accountability across the education, health, agriculture, and local government sectors. [48] In response to these challenges, the Partnership and Advocacy pillar of GREENEDU NEXUS proposes formal governance mechanisms to strengthen coordination among education, health, food security, and local government sectors while clarifying institutional roles and responsibilities. Furthermore, limited family engagement indicates that existing interventions remain predominantly school-centred, underscoring the importance of positioning parents as active partners through culturally appropriate education and communication strategies to support sustainable improvements in children's food safety and nutrition behaviours. This finding reinforces recommendations from whole-school health promotion frameworks, which recognise family and community engagement as essential components for achieving sustained behavioural and public health outcomes. This qualitative needs assessment found that food safety and nutrition education in Indonesian primary schools exists but is fragmented and inconsistently implemented. Integrating insights on curriculum practices, stakeholder roles, and implementation challenges, the study informed the development of the GREENEDU NEXUS model, a five-pillar, whole-school framework combining food safety, nutrition, and green economy principles. By aligning standardised learning content, experiential pedagogy, safe and sustainable canteens, green behaviour, and multi-stakeholder collaboration, the model offers a practical and policy-relevant approach to strengthening school-based food safety and nutrition education. Future research should focus on pilot testing and rigorous evaluation to assess effectiveness, scalability, and long-term impacts across diverse school contexts.

### 4.1 Implications for research, practice, and policy

The findings of this study have important implications for research, practice, and policy. Future research should move beyond needs assessment towards pilot implementation and rigorous evaluation of integrated models such as GREENEDU NEXUS using mixed-methods and longitudinal designs to examine their effects on student behaviours, school food environments, and health outcomes across diverse educational contexts. From a policy perspective, institutionalising food safety and nutrition education within formal curricula, teacher education, and school food governance through competency-based learning modules, minimum infrastructure standards, and structured multi-sectoral coordination may help reduce variability in implementation and strengthen long-term sustainability. In practice, the findings suggest that improving food safety and nutrition education requires a shift from prohibition-based approaches towards behavioural empowerment supported by enabling school food environments, including experiential learning, safe and sustainable canteen management, family engagement, and partnerships with local food systems. Such integrated approaches have the potential to strengthen children's food literacy while promoting healthier school food environments and supporting broader public health goals. This study is strengthened by its qualitative design, which captured diverse stakeholder perspectives across education, health, food security, and policy sectors and integrated behavioural theory with Sustainable Development Goal principles to inform the development of the GREENEDU NEXUS model. Nevertheless, the findings are context-specific and primarily reflect stakeholder perspectives rather than direct behavioural outcomes. In addition, informal food environments surrounding schools were not explored comprehensively, and the proposed model has not yet undergone pilot implementation. Future implementation studies are therefore needed to evaluate its effectiveness, scalability, and long-term contribution to school-based food safety, nutrition, and public health.

## Ethical statement

Ethical approval was obtained from the Ethics Committee of Universitas Muhammadiyah Surakarta (approval number: 249/KE/04/2025). All participants provided written informed consent after receiving detailed information regarding the study objectives, procedures, voluntary participation, and confidentiality. Participants were informed of their right to withdraw from the study at any stage without any consequences. Data confidentiality was ensured through the anonymization of transcripts and secure storage of all research materials.

## Funding

This research was supported by a Riset Kolaborasi Indonesia (RKI) 2025 research grant through the 10.13039/501100014823Directorate of Research and Community Service, 10.13039/501100005115Universitas Pendidikan Indonesia, under Grant No. 4551 /UN40.A5/PT.01.03/2025. The funding body had no role in the study design, data collection, data analysis, interpretation of the findings, or preparation of the manuscript.

## AI statement

During the preparation of this work, the authors used ChatGPT solely for English language improvement, including grammar and spelling checking, paraphrasing of individual sentences, and improving clarity and readability. The scientific content, ideas, analysis, interpretation, argumentation, and overall structure of the manuscript were developed and determined by the authors. After using the tool, the authors reviewed and edited the content as needed and take full responsibility for the content of the published article

## Declaration of competing interest

The authors declare no competing interests.
